# Supplementary material for: A novel brain partition highlights the modular skeleton shared by structure and function
Source: Sci Rep. 2015 Jun 3;5:10532. doi: 10.1038/srep10532 (PMC4453230; doi:10.1038/srep10532)
Supplement: Supplementary Information [file srep10532-s1.doc]

**Supplementary Information:**

**A novel brain partition highlights the modular skeleton**

**shared by structure and function**

Ibai Diez^1,*^, Paolo Bonifazi^2,*^, Iñaki Escudero^1,3^, Beatriz Mateos^1,3^, Miguel A. Muñoz^4^, Sebastiano Stramaglia^1,5,6,**,§^ and Jesus M Cortes^1,6,7,§^

1. Biocruces Health Research Institute, Cruces University Hospital, Barakaldo, Spain.

2. School of Physics and Astronomy, George S. Wise Faculty of Life Sciences, Sagol School of Neuroscience, Tel Aviv University, Tel Aviv, Israel.

3. Radiology Service, Cruces University Hospital, Barakaldo, Spain.

4. Departamento de Electromagnetismo y Física de la Materia and Instituto Carlos I de Física Teórica y Computacional, Universidad de Granada, Spain.

5. Dipartimento di Fisica, Universita degli Studi di Bari and INFN, Bari, Italy.

6. Ikerbasque: The Basque Foundation for Science, Bilbao, Spain.

7. Department of Cell Biology and Histology. University of the Basque Country. Leioa, Spain

* These authors contributed equally to this work.

** New address: BCAM - Basque Center for Applied Mathematics, Bilbao, Spain

§ To whom correspondence should be addressed.

Email: [Sebastiano.Stramaglia@ba.infn.it](mailto:Sebastiano.Stramaglia@ba.infn.it) and [jesus.cortesdiaz@osakidetza.net](mailto:jesus.cortesdiaz@osakidetza.net)

**Table S1: Anatomical description of the 20 modules defined after hierarchical agglomerative clustering (HAC) of rsFC.** In the first column, we also provide the module volume and links to the 3D movies are given in the third column.

| **Module number (volume size)** | **Anatomical description** | **Link to 3D representation** |
| --- | --- | --- |
| module 1  (7.26 cm^3^) | **Posterior cingulate:** posterior area of the cingulate gyrus or callosal convolution. Located above the corpus callosum, it goes from the frontal lobe back to the temporal uncus and up to the splenium. It belongs to the Default Mode Network. | Movie S1 |
| module 2  (104.36 cm^3^) | **Putamen:** a round structure located at the base of the telencephalon. It is also one of the basal ganglia structures.  **Anterior cingulate:** anterior frontal region of the cingulate gyrus, initiated above the rostrum of the corpus callosum.  **Rostral pars of the middle frontal gyrus:** anterior inferior end of the middle frontal gyrus.  **Superior parietal gyrus:** parietal gyrus located posterior to the postcentral gyrus.  **Supramarginal gyrus**: region in the parietal lobe encircling the posterior extreme of the Sylvian fissure.  **Insula:** triangular area of cerebral cortex forming the medial wall of the Sylvian fissure.  **Inferior parietal gyrus:** parietal gyrus located behind the postcentral gyrus and below the superior parietal gyrus.  **Precentral gyrus:** frontal gyrus that defines the anterior boundary of the fissure of Rolando with a mainly motor function.  **Superior frontal gyrus:** antero-superior parasagittal frontal gyrus, located anterior to the precentral gyrus. | Movie S2 |
| module 3  (221.18 cm^3^) | **Paracentral lobule:** medial gyrus that connects the pre- and postcentral gyrus.  **Precentral gyrus** (cf. Module 2)  **Postcentral gyrus:** Parietal gyrus located between the fissure of Rolando and the postcentral sulcus, which has a mainly sensory function.  **Precuneus:** square brain lobule located before the parietal-occipital sulcus and behind the paracentral lobule at the medial surface of the brain hemisphere.  **Superior frontal gyrus** (cf. module 2).  **Superior parietal gyrus** (cf. module 2)  **Superior temporal gyrus:** temporal gyrus at the lateral surface of the temporal lobe. It is located below the Sylvian fissure and above the superior temporal sulcus. It belongs to the temporal neocortex.  **Supramarginal gyrus** (cf. module 2).  **Insula** (cf. module 2) | Movie S3 |
| module 4  (91.48 cm^3^) | **Cuneus:** occipital gyrus between the parieto-occipital sulcus and the calcarine sulcus at the medial surface of the occipital lobe.  **Lateral occipital sulcus:** external lateral surface of the occipital lobe close to the occipital lobe, dividing the external occipital gyrus.  **Lingual gyrus:** occipital extension of the parahippocampal gyrus at the medial surface of the occipital lobe.  **Pericalcarine cortex:** occipital area encircling the calcarine sulcus with a function associated to visual perception.  **Precuneus** (cf. module 3) | Movie S4 |
| module 5  (37.02 cm^3^) | **Medial frontal gyrus:** frontal gyrus at the lateral surface below the superior frontal gyrus.  **Precentral gyrus** (cf. module 2)  **Rostral pars of the middle frontal gyrus** (cf. module 2) | Movie S5 |
| module 6  (159.33 cm^3^) | **Cerebellum:** posterior part of the rombencephalon made up of the two hemispheres and the central vermis. It is located below the occipital lobe.  **Fusiform gyrus:** temporal gyrus in the inferior surface between the inferior temporal gyrus and the parahippocampal gyrus. It has two areas, the medial occipito-temporal gyrus and the lateral occipito-temporal gyrus.  **Inferior temporal gyrus:** inferior gyrus located in the lateral surface of the temporal lobe, below the inferior temporal sulcus.  **Lateral occipital sulcus** (cf. module 4)  **Superior parietal gyrus** (cf. module 2) | Movie S6 |
| module 7  (22.30 cm^3^) | **Thalamus:** middle symmetrical structure of the diencephalon with multiple afferent and efferent connections, situated around the third ventricle.  **Caudate nucleus** (symmetrical structure): one of the basic structures of the basal ganglia belonging to the corpus striatum. It is located at the lateral surface of the lateral ventricles surrounding the thalamus.  **Putamen** (cf. module 2)  **Pallidum:** symmetrical structure within the basal ganglia. Medial diencephalic region of the lenticular nucleus.  **Accumbens nucleus:** symmetrical structure located in the ventral region of the corpus striatum, therefore belonging to the basal ganglia. | Movie S7 |
| module 8  (3.29 cm^3^) | **Caudate nucleus** (cf. module 7)  **Putamen** (cf. module 2) | Movie S8 |
| module 9  (163.67 cm^3^) | **Cerebellum** (cf. module 6)  **Caudal middle frontal:** frontal gyrus on the lateral surface, located below and lateral to the superior frontal gyrus. This region refers to its most caudal part.  **Cingulate isthmus:** intersection narrowing between the cingulate and the hippocampal gyrus. It is located behind and below the splenium of corpus callosum.  **Posterior cingulate** (cf. module 1)  **Precuneus** (cf. module 3)  **Inferior parietal gyrus** (cf. module 2)  **Rostral pars of the middle frontal gyrus** (cf. module 2)  **Superior frontal gyrus** (cf. module 2) | Movie S9 |
| module 10  (103.55 cm^3^) | **Anterior cingulate** (cf. module 2)  **Inferior parietal gyrus** (cf. module 2)  **Orbital gyrus:** frontobasal gyrus lateral located to the straight gyrus.  **Pars opercularis:** opercular part of the inferior frontal gyrus.  **Pars orbitalis:** orbital part of the inferior frontal gyrus.  **Pars triangularis:** inferior part of the inferior frontal gyrus.  **Anterior cingulate** (cf. module 2)  **Rostral pars of middle frontal gyrus** (cf. module 2)  **Superior frontal gyrus** (cf. module 2) | Movie S10 |
| module 11  (31.08 cm^3^) | **Caudate nucleus** (cf. module 7)  **Accumbens nucleus** (cf. module 7)  **Lateral frontal orbital gyrus:** external orbital gyrus, located frontobasal and lateral to the medial orbitofrontal gyrus.  **Orbital gyrus** (cf. module 10)  **Anterior cingulate** (cf. module 10) | Movie S11 |
| module 12  (33.24 cm^3^) | **Inferior parietal gyrus** (cf. module 2)  **Inferior temporal gyrus** (cf. module 6)  **Lateral frontal orbital gyrus** (cf. Module 11)  **Pars orbitalis** (cf. module 10)  **Pars triangularis** (cf. module 10)  **Rostral pars of the middle frontal gyrus** (cf. module 2)  **Superior frontal gyrus** (cf. module 2)  **Caudate nucleus and anterior cingulate** (cf. module 7 and module 2) | Movie S12 |
| module 13  (24.46 cm^3^) | **Middle frontal gyrus:** caudal part of the middle frontal gyrus.  **Pars opercularis** (cf. module 10)  **Precentral gyrus** (cf. module 2)  **Superior frontal gyrus** (cf. module 2) | Movie S13 |
| module 14  (92.75 cm^3^) | **Thalamus** (cf. module 7)  **Hippocampus:** symmetrical grey matter structure, located in the mesial-temporal region, at the base of the temporal horn.  **Amygdala:** grey nuclei located in the temporal uncus, above the temporal ventricular horn. It belongs to the rhinencephalon.  **Putamen** (cf. modulo 2)  **Ventral diencephalon:** multiple structures containing the hypothalamus, mammillary tubercle, subthalamic nucleus, substantia nigra, red nucleus, geniculate body, optic tract and cerebral peduncles.  **Banks of the superior temporal sulcus:** Temporal lobe structure between the superior temporal gyrus and the middle temporal gyrus.  **Parahippocampal gyrus:** convolution located below the hippocampal sulcus in the temporal mesial region.  **Superior temporal gyrus** (cf. module 3)  **Insula** (cf. module 2)  **Middle temporal gyrus:** gyrus located on the lateral surface of the temporal lobe between the inferior and superior temporal sulcus.  **Temporal pole:** anterior end of the temporal lobe. | Movie S14 |
| module 15  (42.96 cm^3^) | **Thalamus** (cf. module 7)  **Putamen** (cf. module 2)  **Pallidum** (cf. module 7)  **Brainstem:** it consists of three parts, the myelencephalon, pons (metencephalon) and midbrain (mesencephalon). It is the main communication route between the brain, spinal cord and peripheral nerves.  **Hippocampus** (cf. module 14)  **Amygdala** (cf. module 14)  **Accumbens nucleus** (cf. module 7)  **Ventral diencephalon** (cf. module 14)  **Orbital gyrus (cf. module 10)**  **Insula** (cf. module 2) | Movie S15 |
| module 16  (65.58 cm^3^) | **Cerebellum** (cf. module 6)  **Banks of the superior temporal sulcus** (cf. module 14)  **Inferior parietal gyrus** (cf. module 2)  **Cingulate isthmus** (cf. module 9)  **Middle temporal gyrus** (cf. module 14)  **Precuneus** (cf. module 3)  **Superior temporal gyrus** (cf. module 3) | Movie S16 |
| module 17  (5.29 cm^3^) | **Banks of the superior temporal sulcus** (cf. module 14)  **Middle temporal gyrus** (cf. module 14) | Movie S17 |
| module 18  (74.39 cm^3^) | **Hippocampus** (cf. module 14)  **Amygdala** (cf. module 14)  **Entorhinal cortex**: area in the medial-temporal lobe located between the hippocampus and temporal neocortex.  **Fusiform gyrus** (cf. module 6)  **Inferior temporal gyrus** (cf. module 6)  **Middle temporal gyrus** (cf. module 14)  **Parahippocampal gyrus** (cf. module 14)  **Temporal pole** (cf. module 14) | Movie S18 |
| module 19  (28.54 cm^3^) | **Cerebellum** (cf. module 6)  **Brainstem** (cf. module 15) | Movie S19 |
| module 20  (34.91 cm^3^ ) | **Cerebellum** (cf. module 6)  **Parahippocampal gyrus** (cf. module 14) | Movie S20 |

A movie with a superposition of the 20 modules can be seen in SI (Movie S21).

**Figure S1:**

**
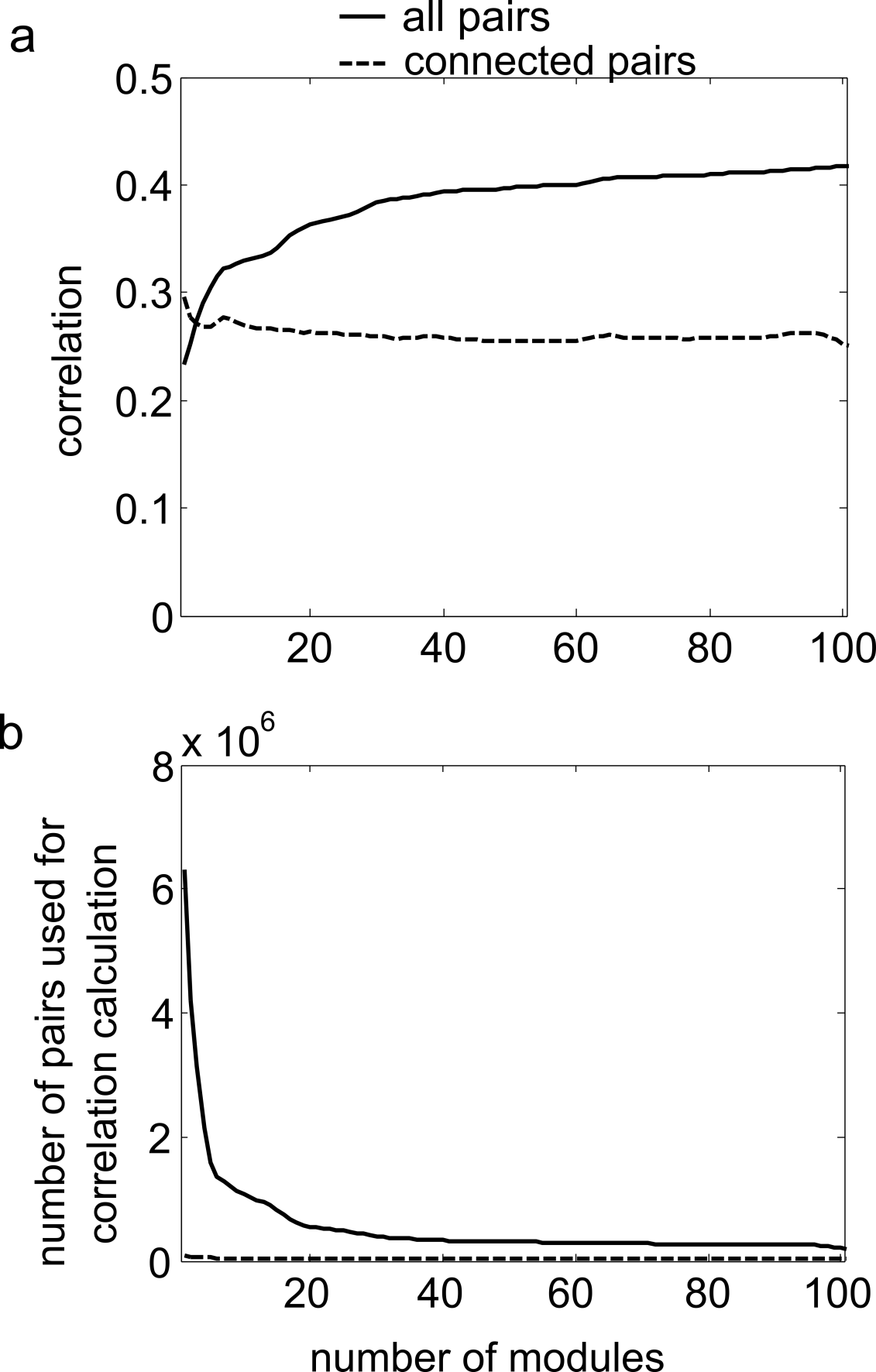
**

**Figure S2:**

**
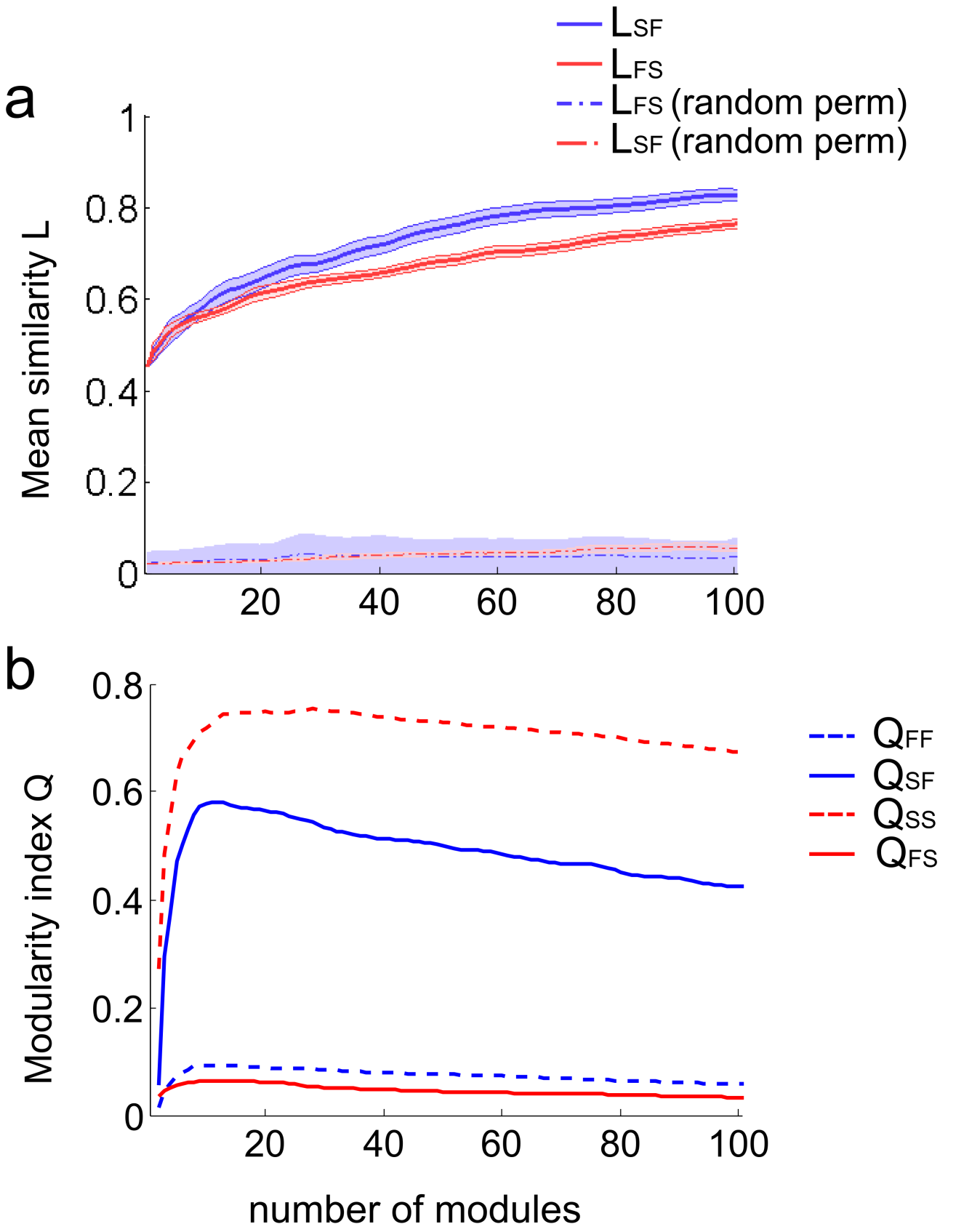
**

**Figure S3:**

**
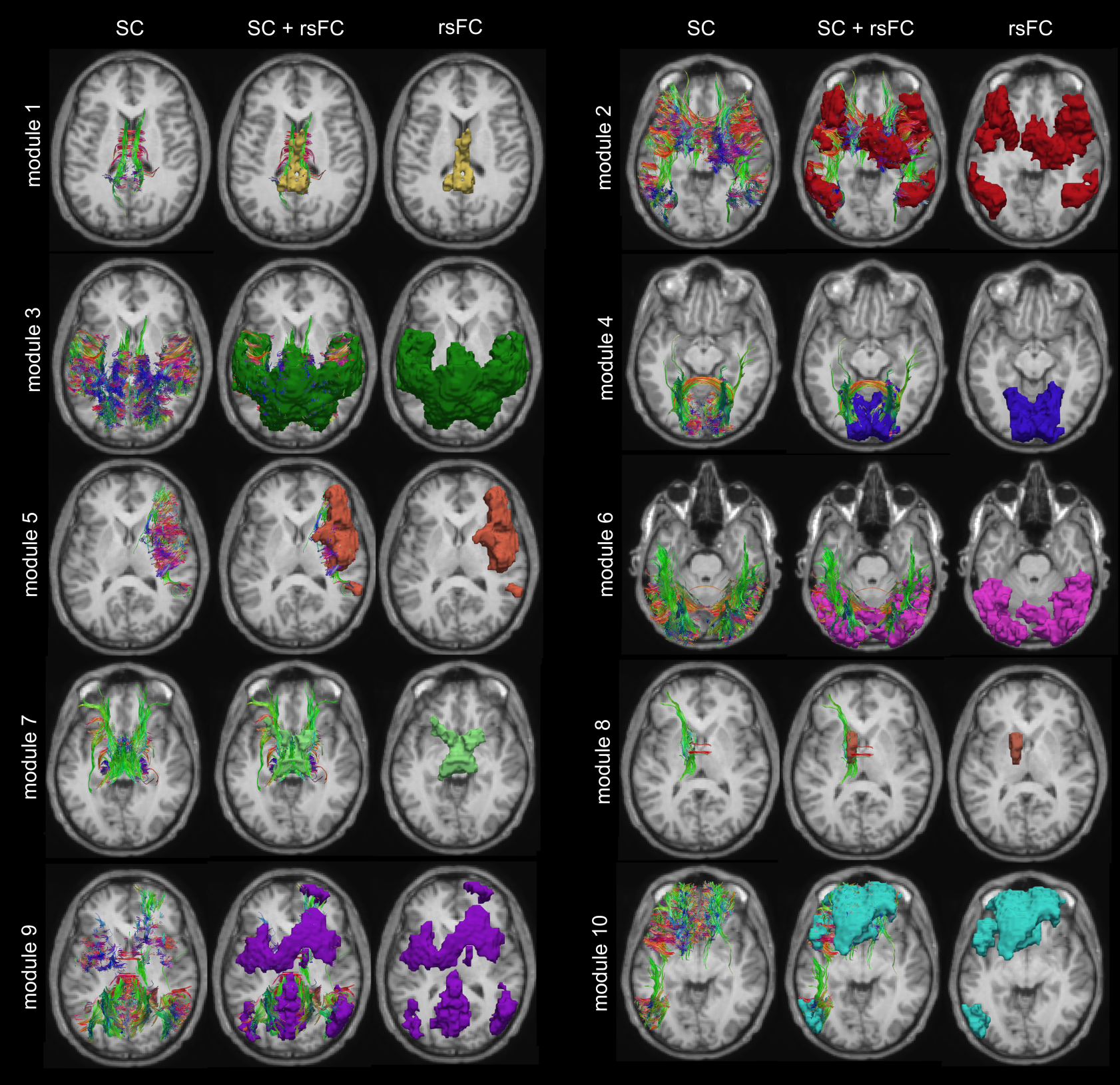
**

**Figure S4:**

**
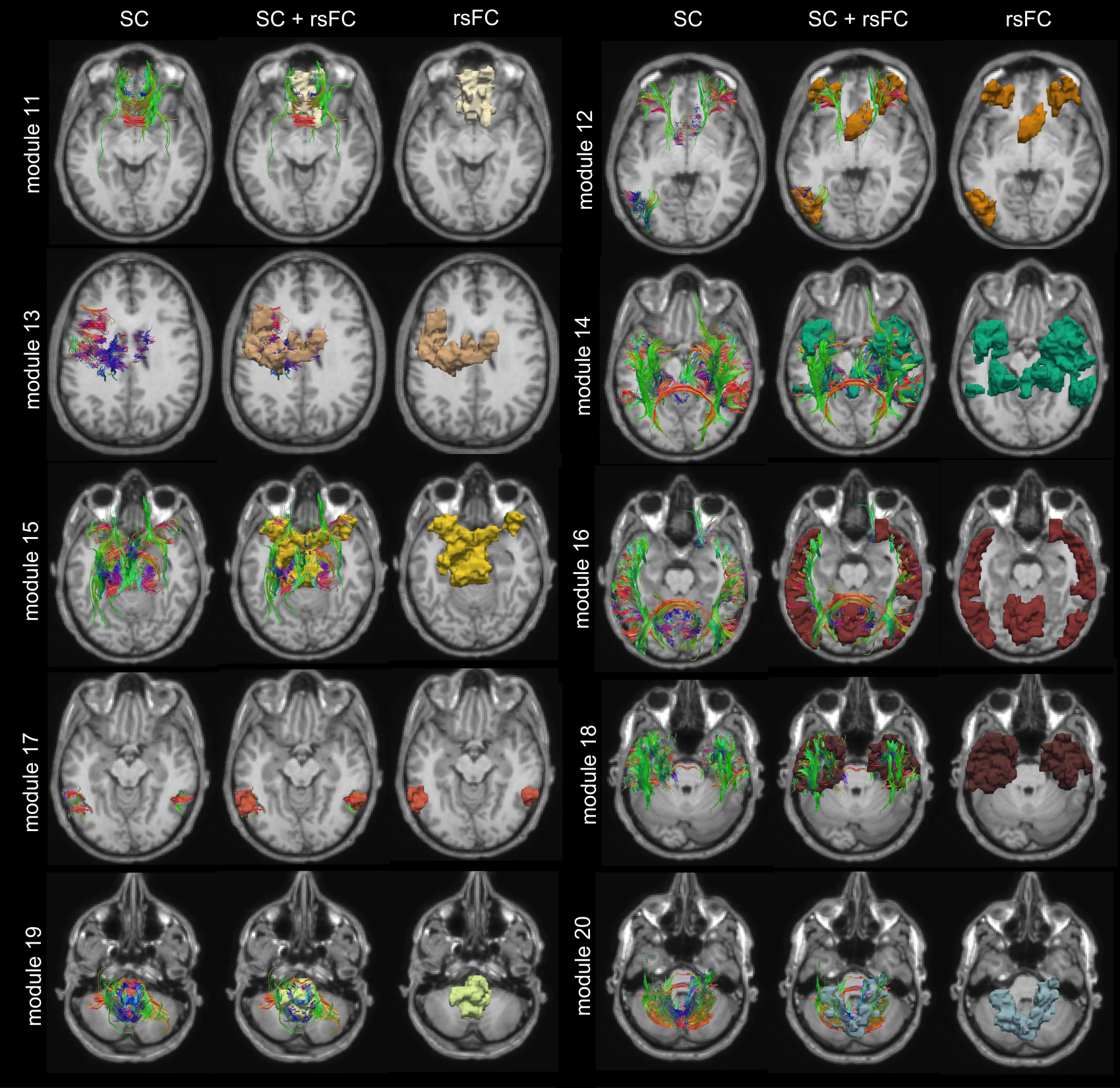
**

**Figure S5:**

**
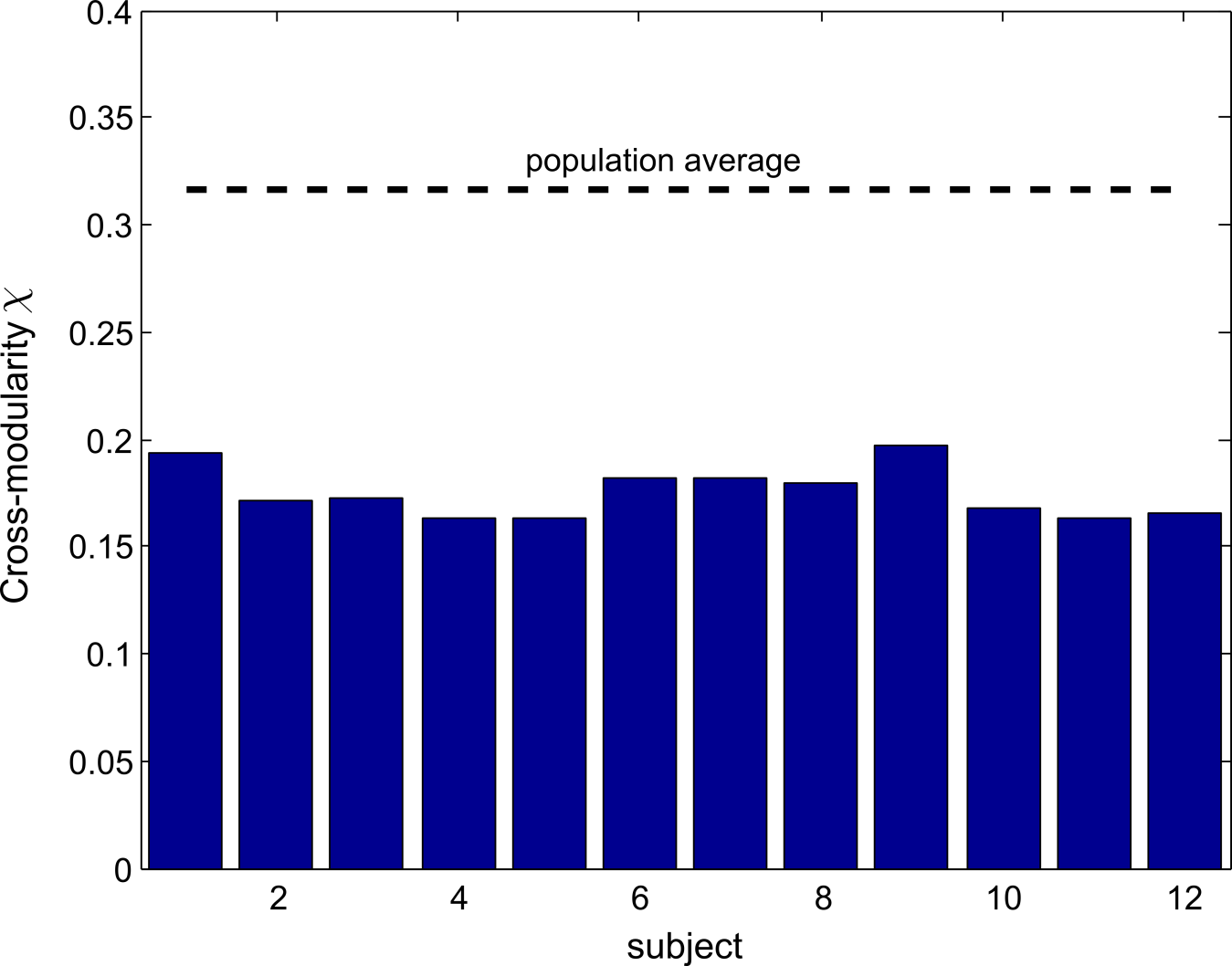
**

**Figure S6:**

**
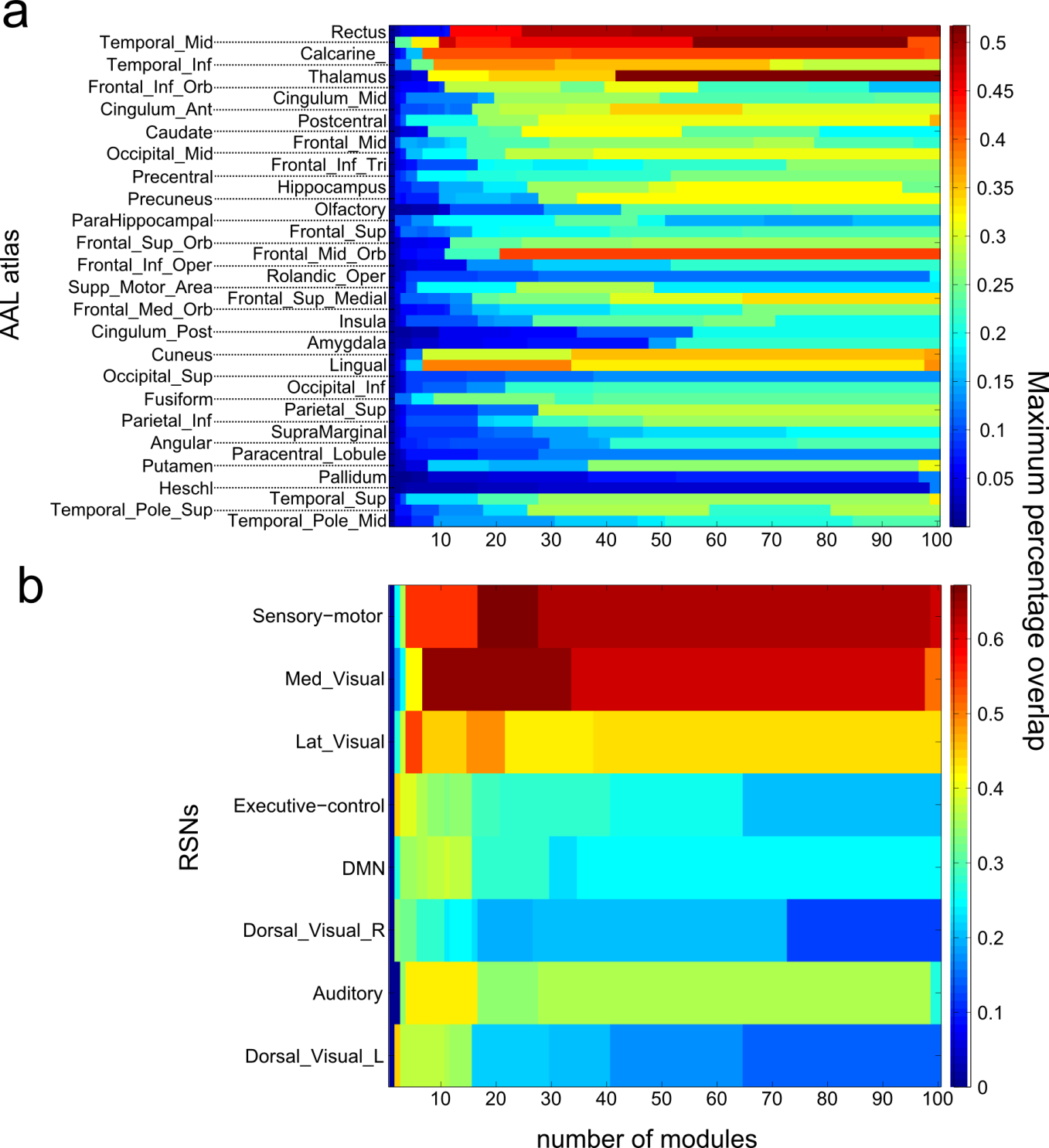
**

**Figure S7:**

**
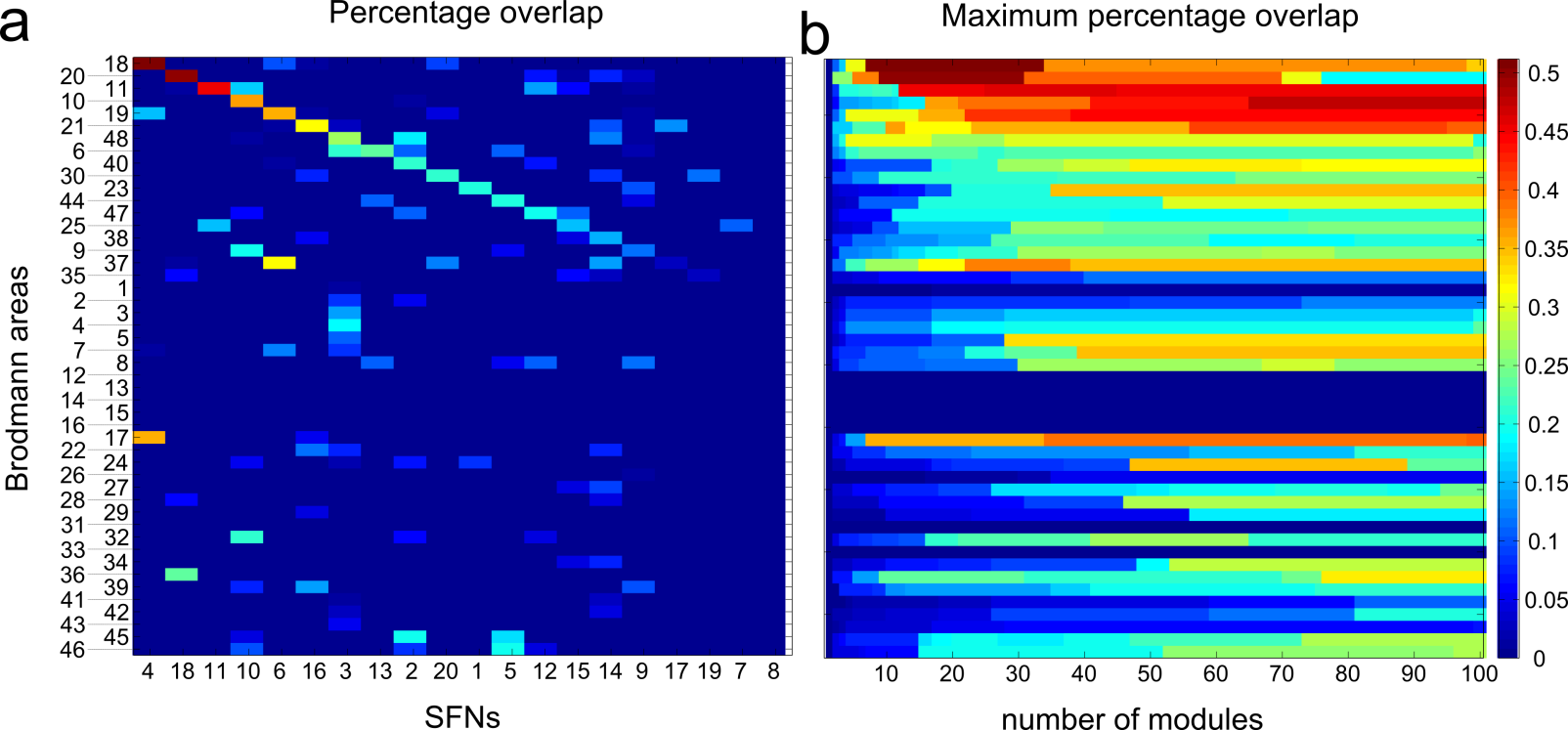
**

**Figure S8:**

**

**

**Figure S9:**

**
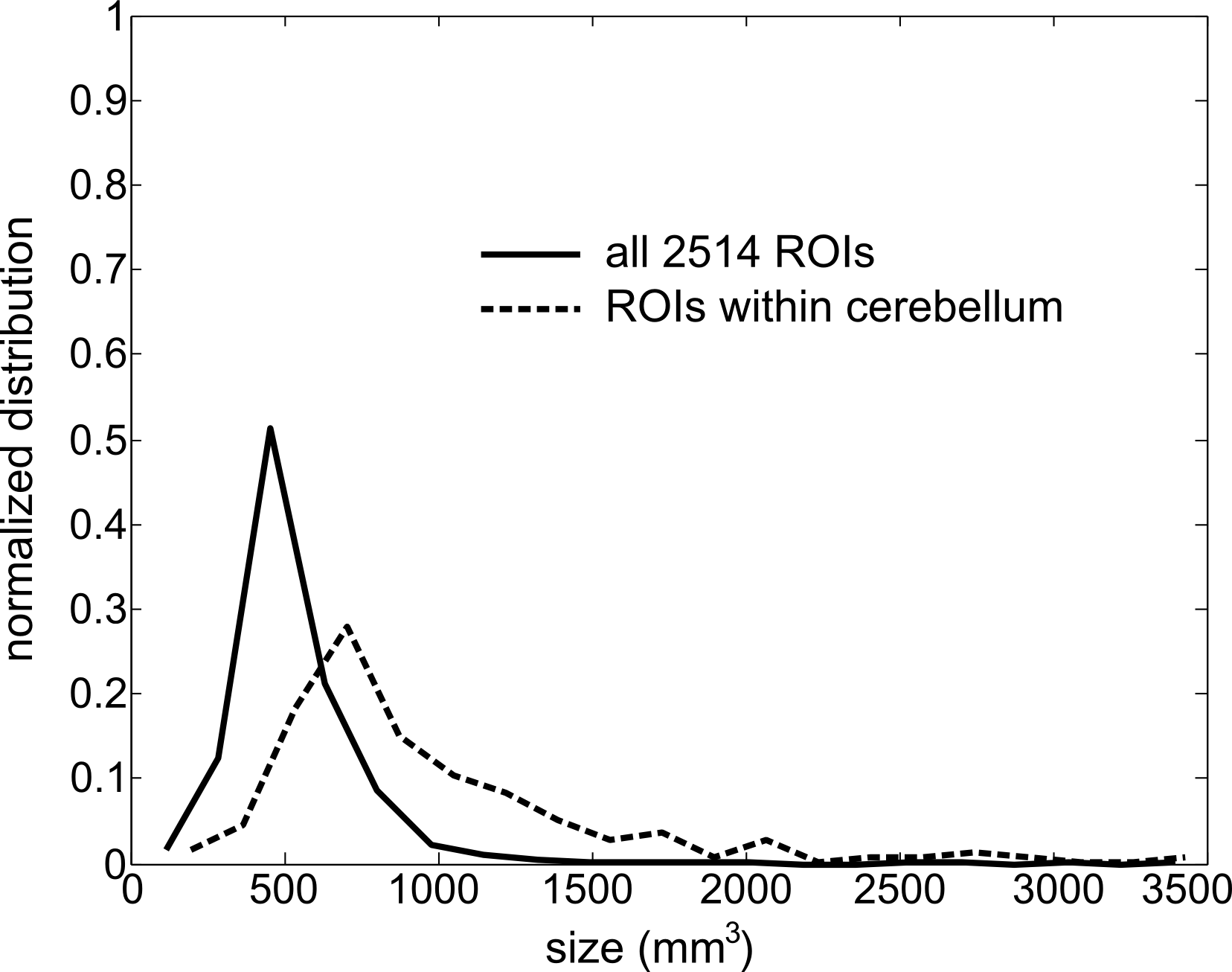
**

**Figure S10:
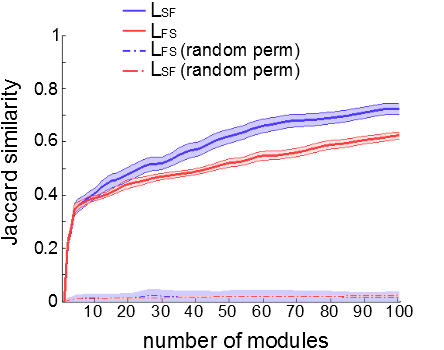
**

**Supplementary Figure Legends**

**Fig. S1: Pearson correlation between rsFC and SC. A:** Similar to the pioneering works on matching rsFC and SC by O. Sporns group, we calculated the Pearson correlation value between rsFC and SC (first calculated using all matrix-elements in rsFC and SC). The situation of a single module in the brain partition recovers that indicated by previous authors. Similarly, for a single module in the brain partition, the correlation calculated on connected pairs (“pairs with fiber number >0”, dashed line) gave a higher value in comparison to all the pairs (solid line). As the number of the modules in the brain partition increases, we represent the correlation between rsFC and SC, yet only on pairs belonging to same moduli in rsFC and SC (after HAC on rsFC, we accordingly reordered the SC). **B:** the number of pairs taken into account for the calculation of the correlation in function of the number of modules.

**Fig. S2: Similarity (L) and Modularity (Q) between rsFC and SC.** **A:** The mean similarity (± SE) between functional and structural moduli was calculated for brain partitions of different sizes varying from 1 (the entire brain) to 100 modules (similarly in panel B). The L_SF_ was obtained by applying hierarchical agglomerative clustering to rsFC and reordering the SC accordingly. The L_FS_ represents the specular case and the dashed lines correspond to the mean similarity in the case of random permutations in rsFC (blue) and SC (red). **B:** Q_FF_ and Q_SF_ refer to the respective modularity of rsFC and SC on the brain modules as achieved by applying HAC to rsFC. Similarly, Q_SS_ and Q_FS_ are the respective modularity of SC and rsFC on the brain modules achieved by applying HAC to SC.

**Fig. S3: SFMs illustrating common modularity structure between SC and rsFC.** From module 1 to 10, we are plotting modules obtained only with SC (left column), with both SC and rsFC (middle column) and only with rsFC (right column).

**Fig. S4: SFMs illustrating common modularity structure between SC and rsFC.** Similar to figure S3 but for modules from 11 to 20.

**Fig. S5: Cross-modularity between rsFC and SC across different subjects.** The dashed line represents the cross-modularity between the average population (n=12) rsFC and SC.

**Fig. S6: Maximum percentage overlap between SFMs and previously described brain parcellations. A:** Maximum percentage overlap between each region in the AAL and all the different SFMs after HAC, varying the number of modules. **B:** similar to A, but for RSNs.

**Fig. S7: Percentage overlap between SFMs and the Brodmann areas.** Similar to figures 6 and S6 but for Brodmann areas.

**Fig. S8: Validation of our results using data from the Human Connectome Project**. Maximization of cross-modularity, on both our data (Bilbao) and WU-Minn Human Connectome Project lead to almost an identical brain partition.

**Fig. S9: Distribution of ROIs’ size.** The solid line corresponds to the size (measured in mm3) distribution for all the 2514 ROIS. The dashed line does the same but for the ROIs located within the cerebellum (a number of 211 to the total 2514 ROIs).

**Fig. S10: Jaccard similarity between SC and rsFC as a function of the number of modules.** The results are very similar to those obtained using Sorensen’s similarity (fig. S2).
