# Supplementary figures and images for: A novel brain partition highlights the modular skeleton shared by structure and function
Source: Sci Rep. 2015 Jun 3;5:10532. doi: 10.1038/srep10532 (PMC4453230; doi:10.1038/srep10532)

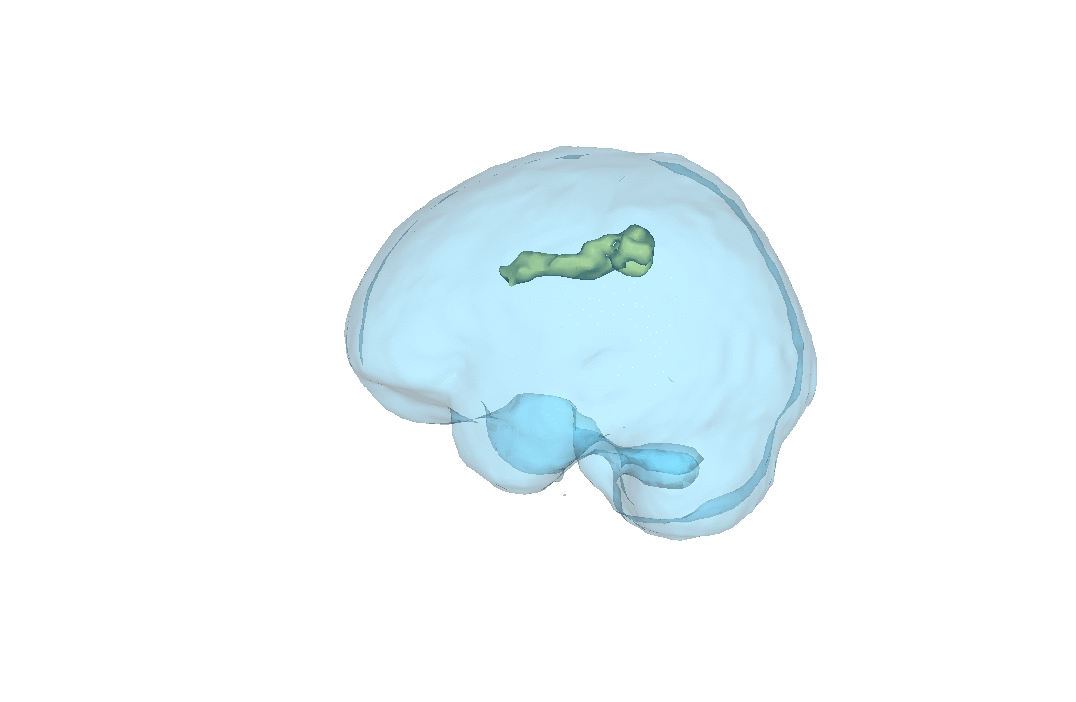

Supplement: Supplementary Movie S1 [file srep10532-s2.gif]

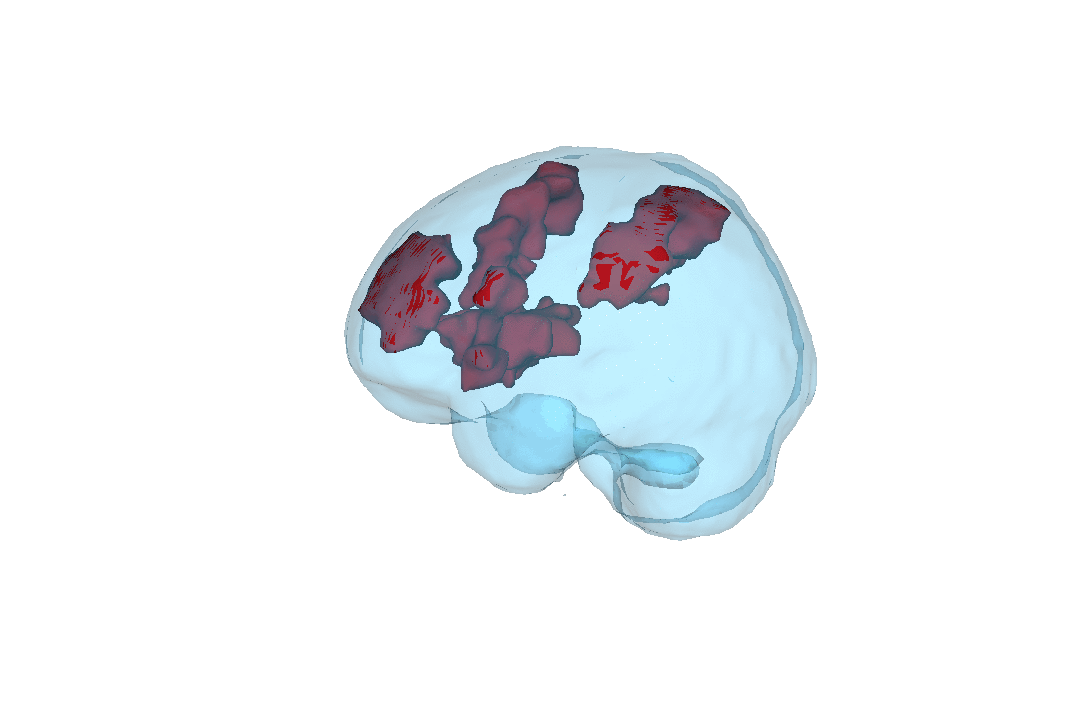

Supplement: Supplementary Movie S2 [file srep10532-s3.gif]

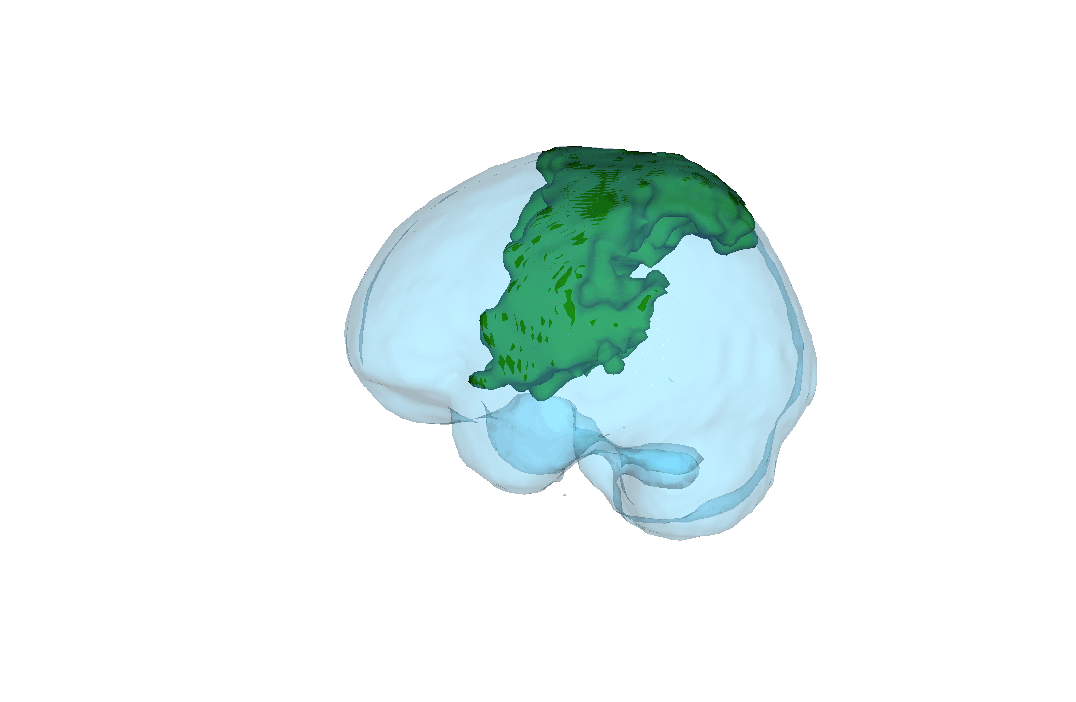

Supplement: Supplementary Movie S3 [file srep10532-s4.gif]

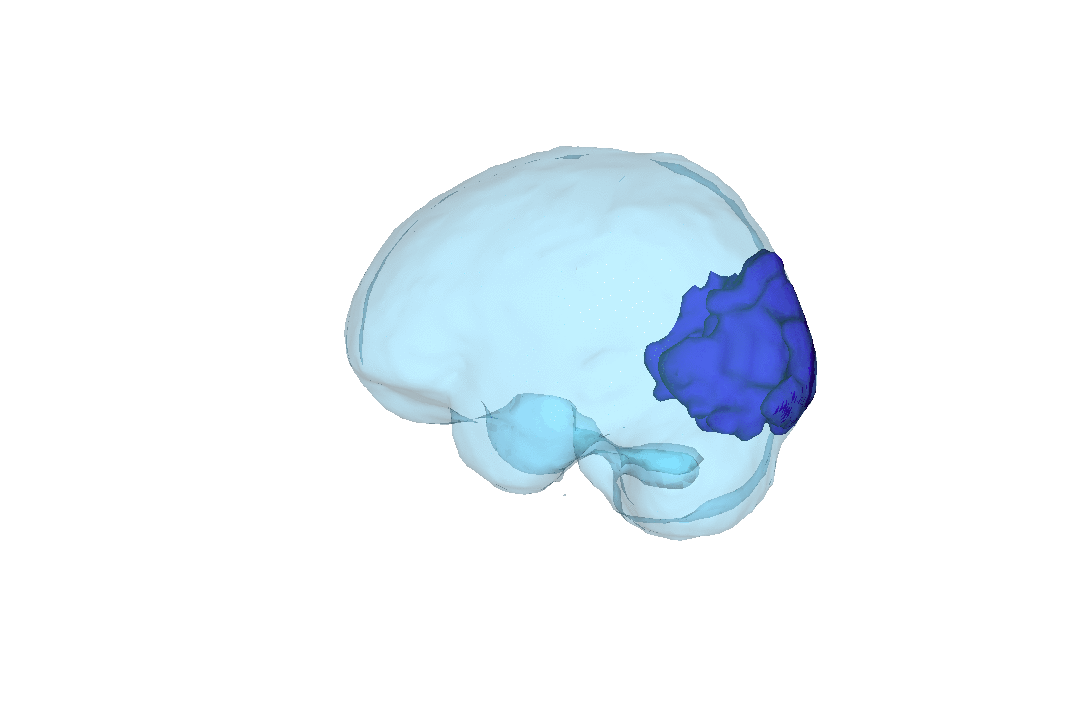

Supplement: Supplementary Movie S4 [file srep10532-s5.gif]

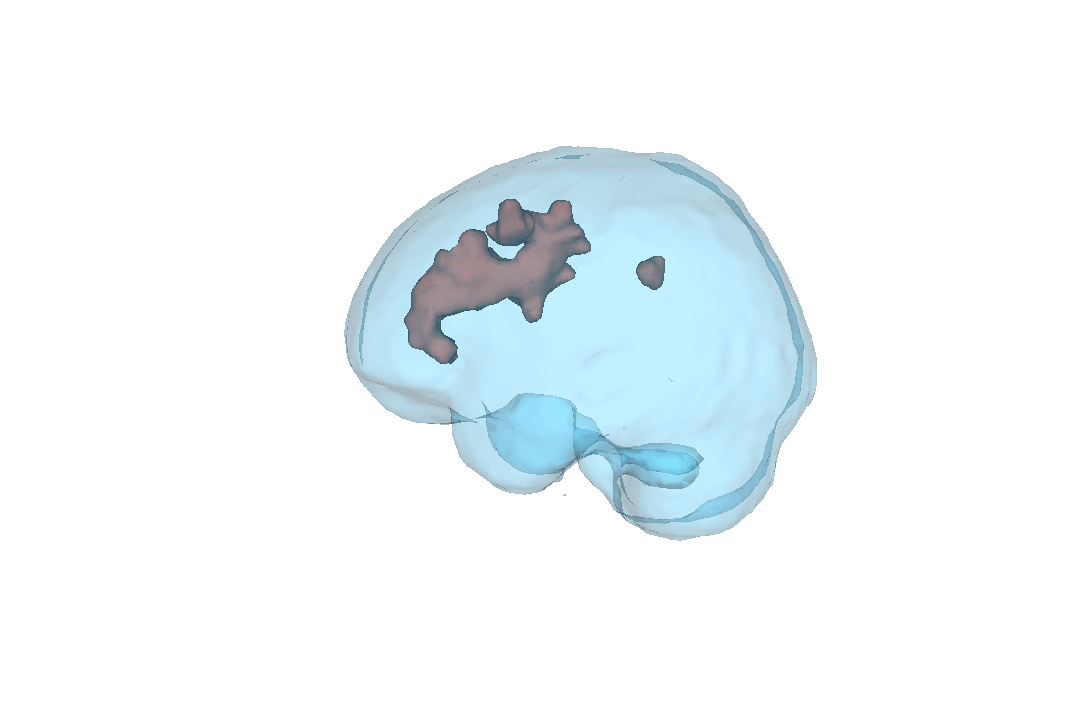

Supplement: Supplementary Movie S5 [file srep10532-s6.gif]

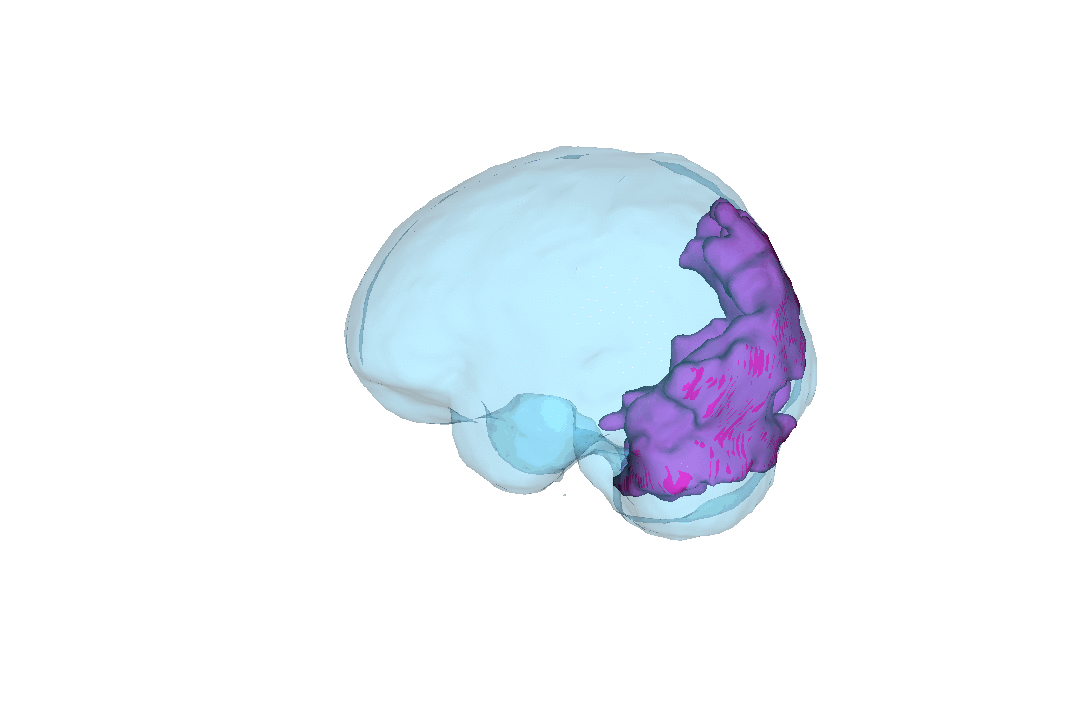

Supplement: Supplementary Movie S6 [file srep10532-s7.gif]

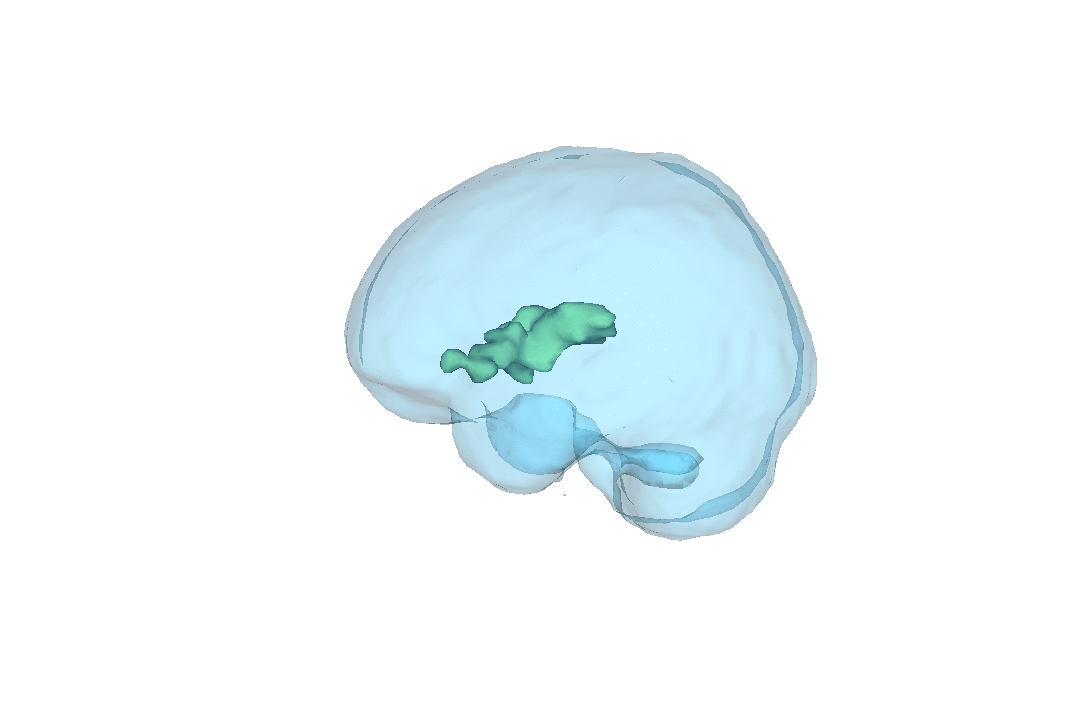

Supplement: Supplementary Movie S7 [file srep10532-s8.gif]

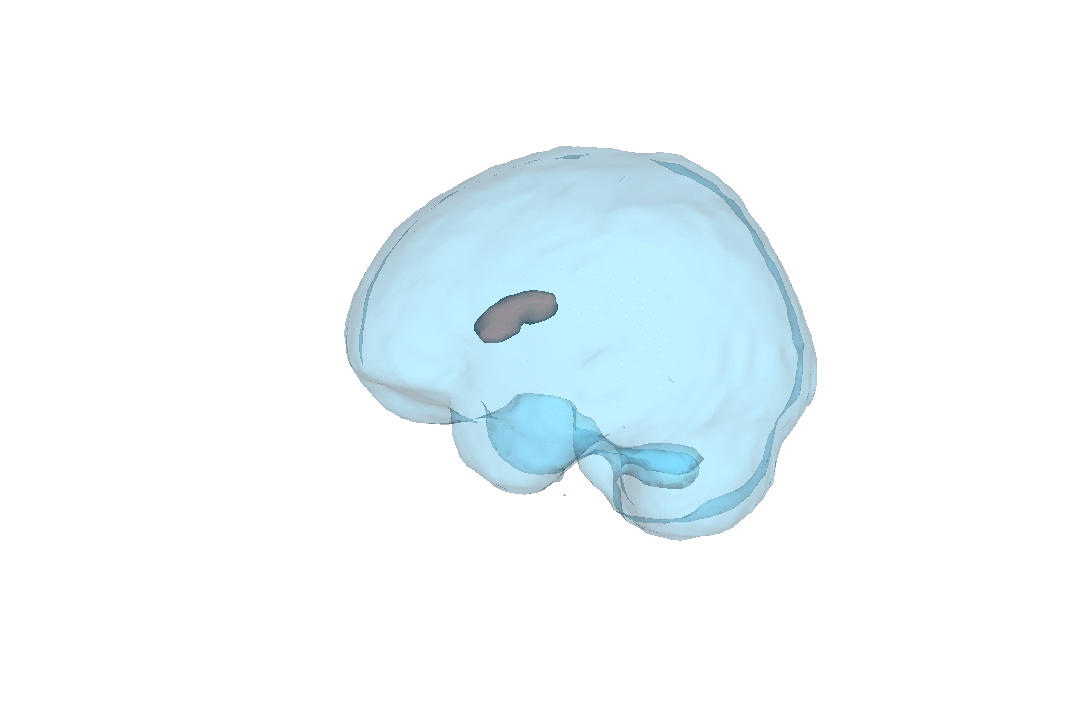

Supplement: Supplementary Movie S8 [file srep10532-s9.gif]

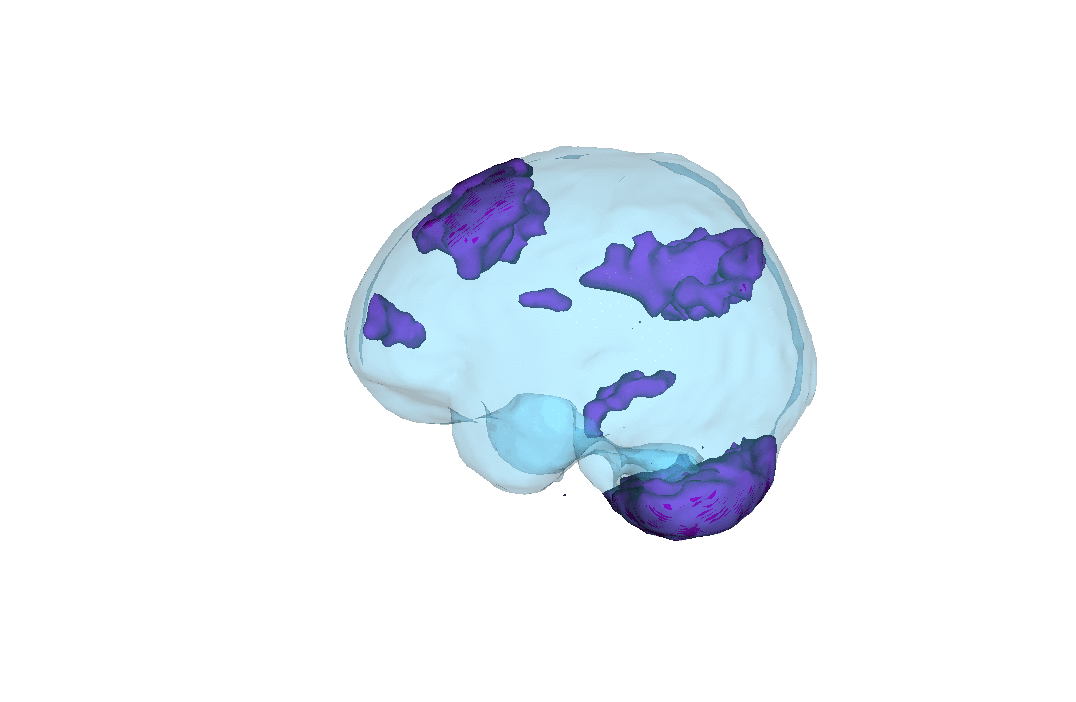

Supplement: Supplementary Movie S9 [file srep10532-s10.gif]

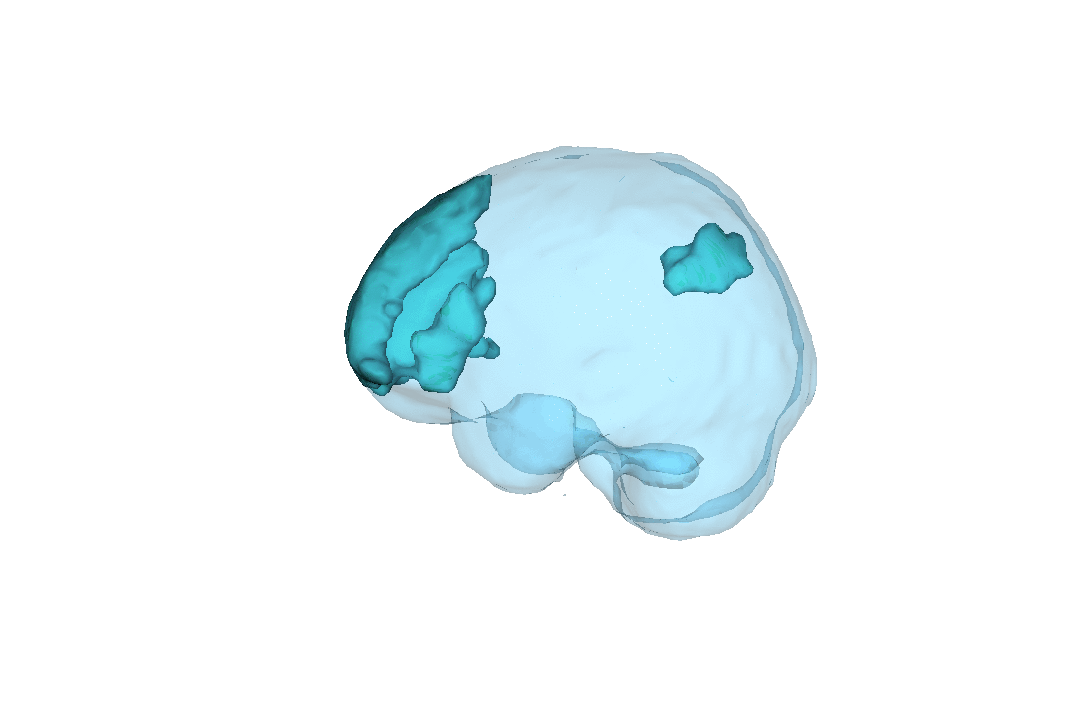

Supplement: Supplementary Movie S10 [file srep10532-s11.gif]

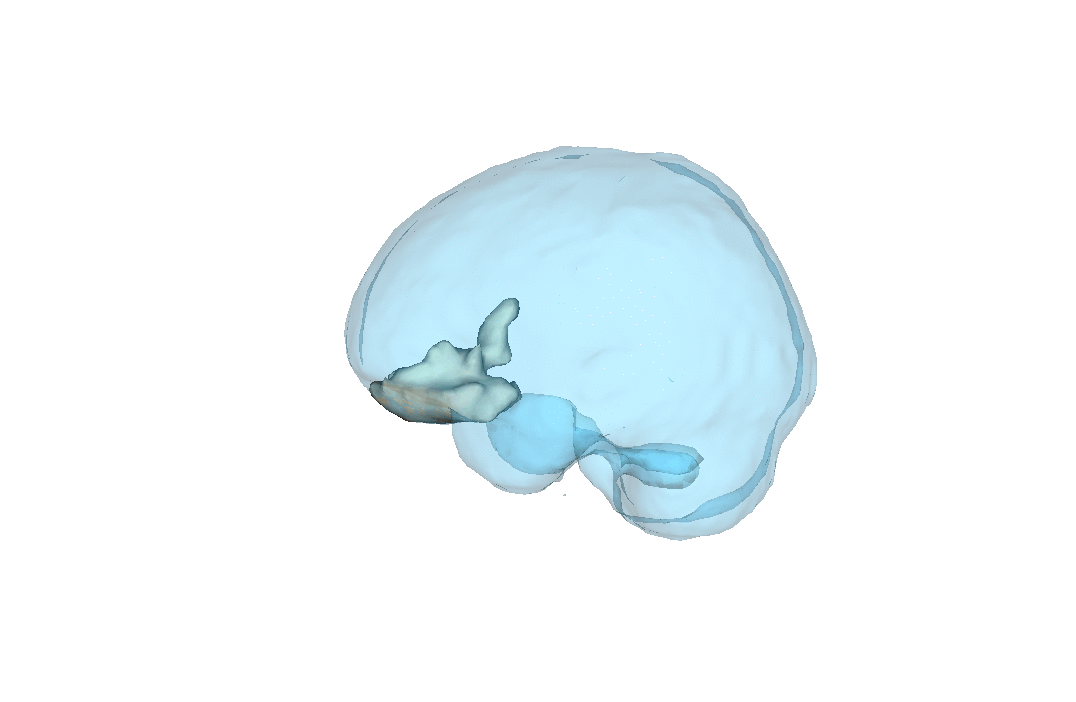

Supplement: Supplementary Movie S11 [file srep10532-s12.gif]

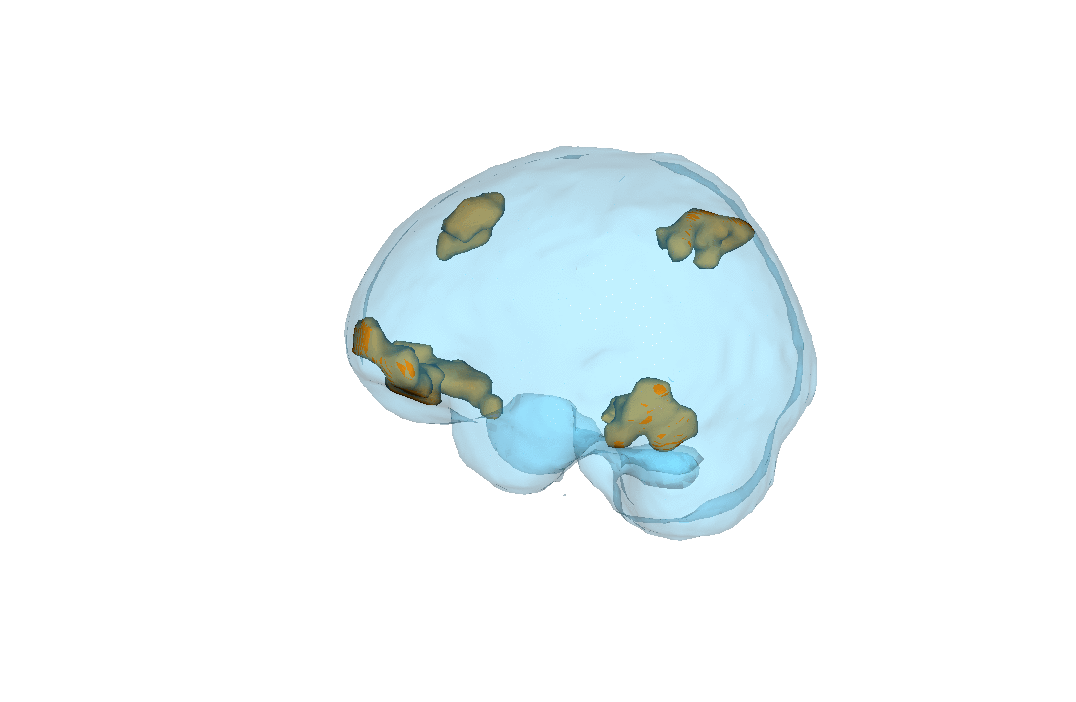

Supplement: Supplementary Movie S12 [file srep10532-s13.gif]

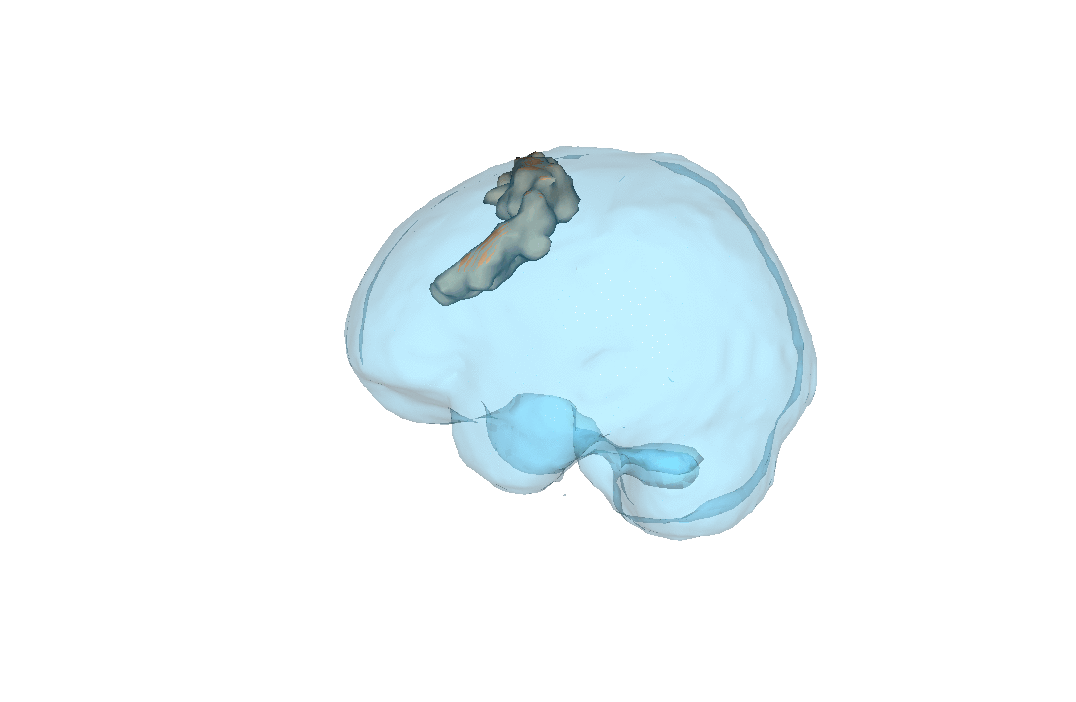

Supplement: Supplementary Movie S13 [file srep10532-s14.gif]

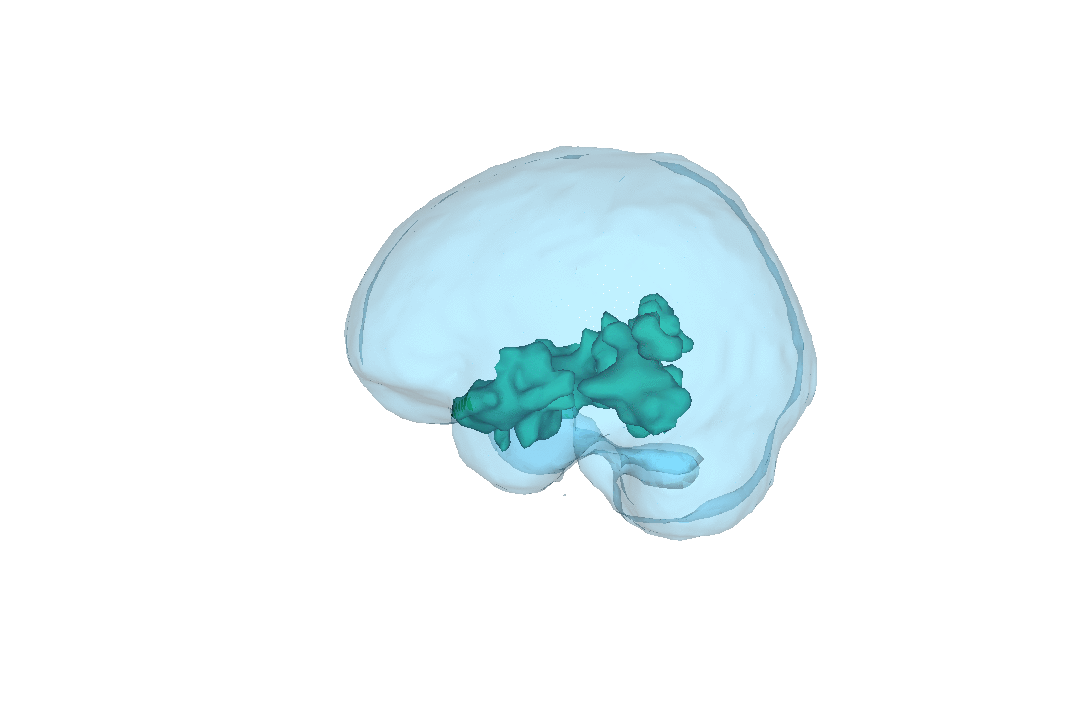

Supplement: Supplementary Movie S14 [file srep10532-s15.gif]

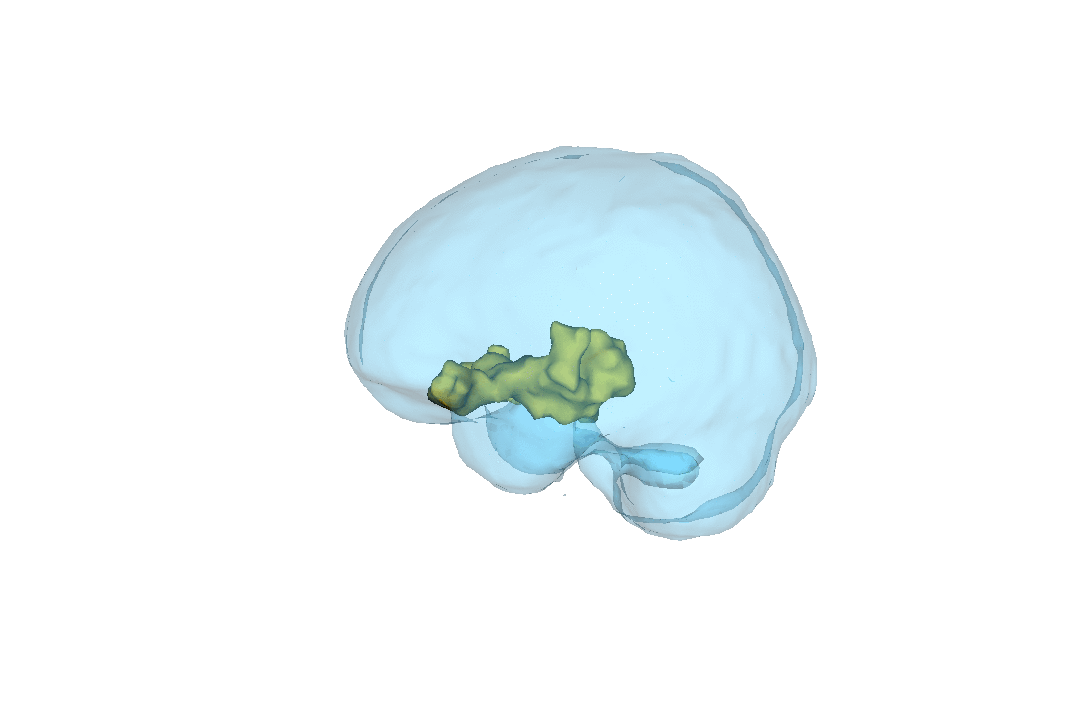

Supplement: Supplementary Movie S15 [file srep10532-s16.gif]

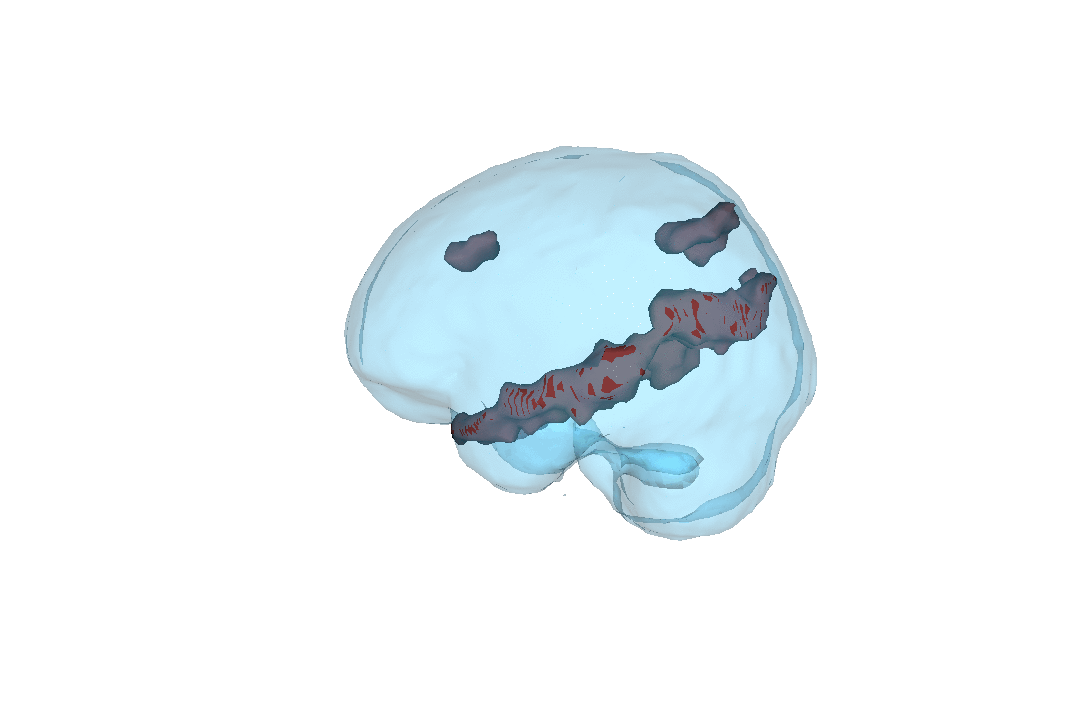

Supplement: Supplementary Movie S16 [file srep10532-s17.gif]

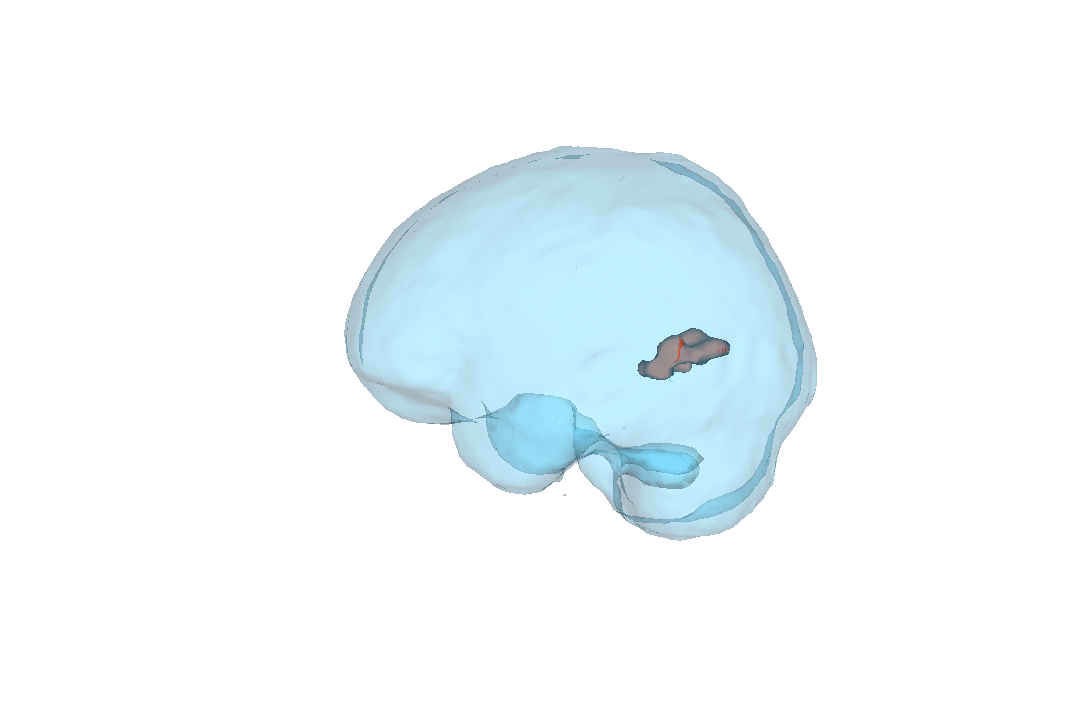

Supplement: Supplementary Movie S17 [file srep10532-s18.gif]

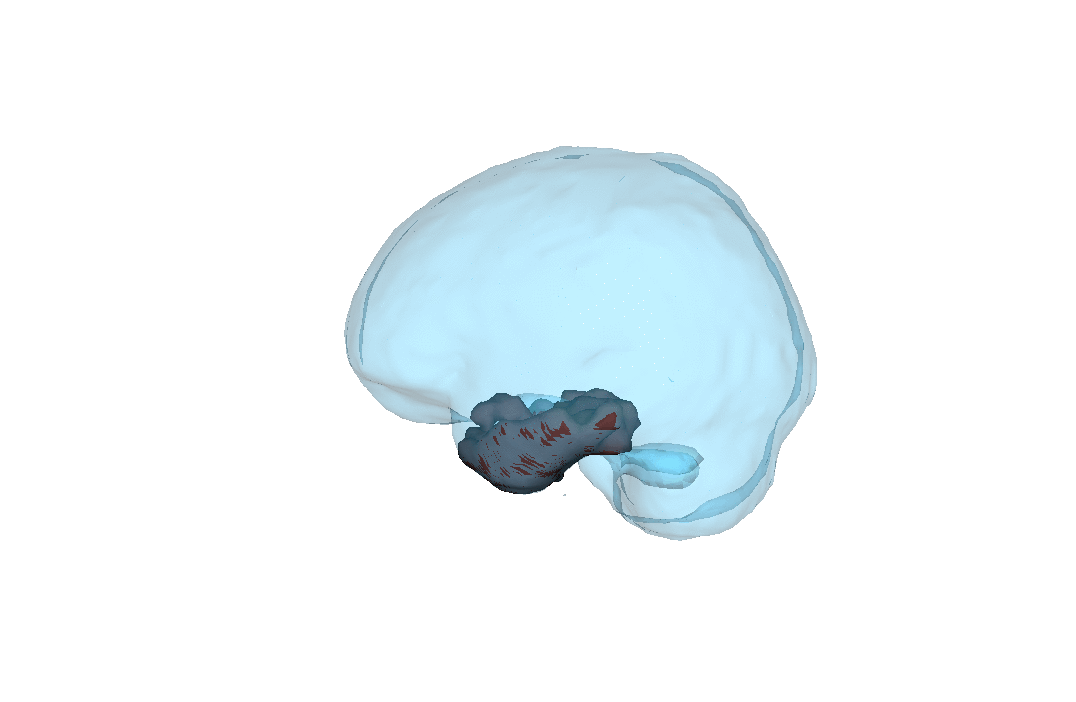

Supplement: Supplementary Movie S18 [file srep10532-s19.gif]

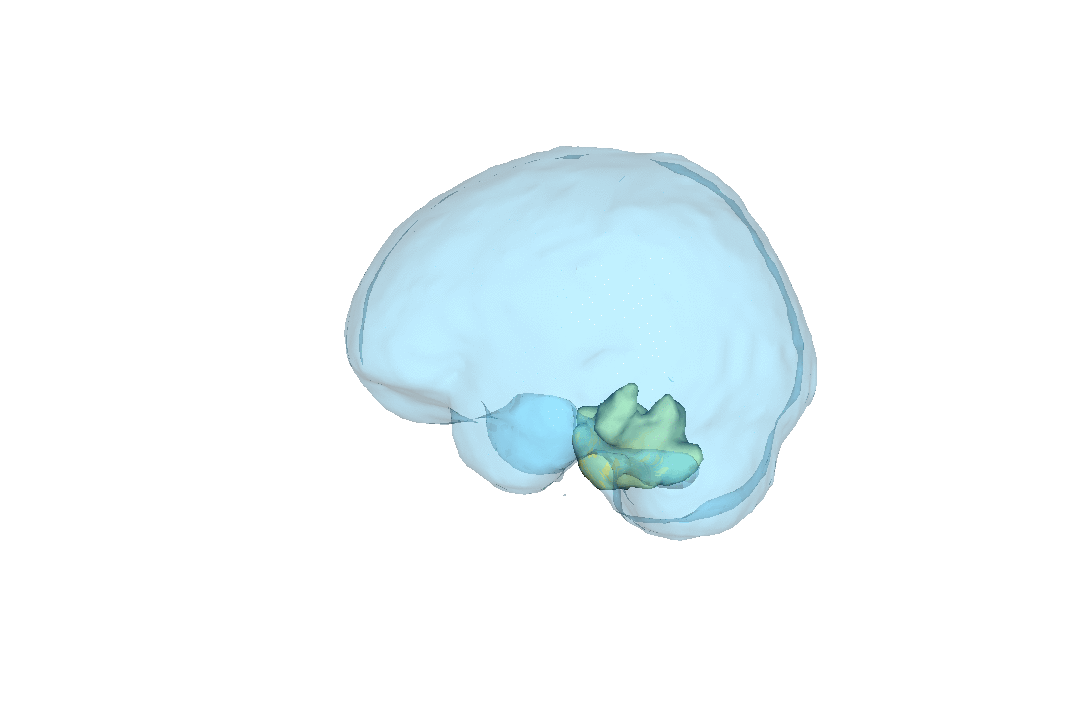

Supplement: Supplementary Movie S19 [file srep10532-s20.gif]

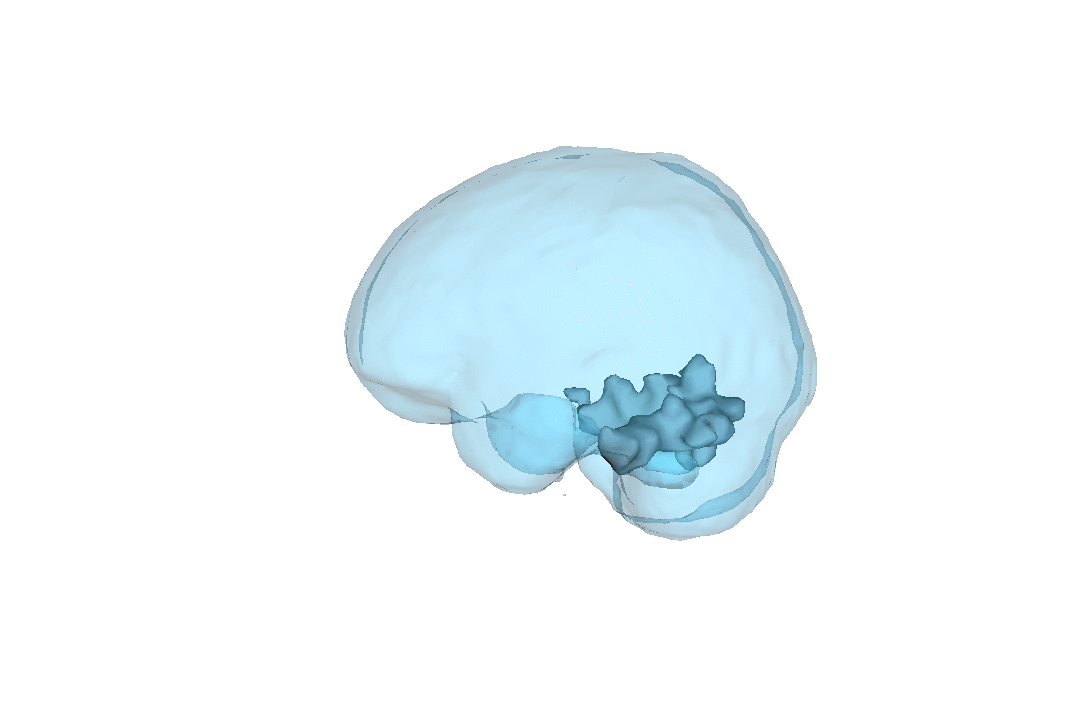

Supplement: Supplementary Movie S20 [file srep10532-s21.gif]

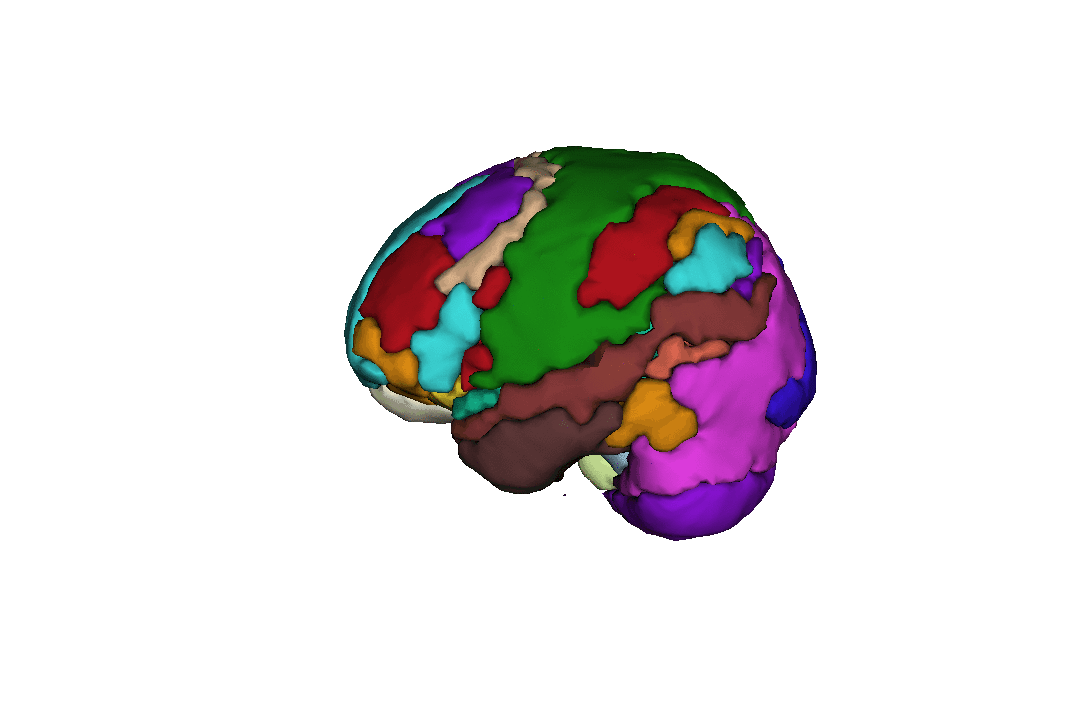

Supplement: Supplementary Movie S21 [file srep10532-s22.gif]
